# Identifying Gut Microbiota Associated With Colorectal Cancer Using a Zero-Inflated Lognormal Model

**DOI:** 10.3389/fmicb.2019.00826

**Published:** 2019-04-24

**Authors:** Dongmei Ai, Hongfei Pan, Xiaoxin Li, Yingxin Gao, Gang Liu, Li C. Xia

**Affiliations:** ^1^Basic Experimental of Natural Science, University of Science and Technology Beijing, Beijing, China; ^2^School of Mathematics and Physics, University of Science and Technology Beijing, Beijing, China; ^3^Department of Medicine, Stanford University School of Medicine, Stanford, CA, United States

**Keywords:** gut microbiota, colorectal cancer, zero-inflated lognormal model, association network, microbial diversity

## Abstract

Colorectal cancer (CRC) is the third most common cancer worldwide. Its incidence is still increasing, and the mortality rate is high. New therapeutic and prognostic strategies are urgently needed. It became increasingly recognized that the gut microbiota composition differs significantly between healthy people and CRC patients. Thus, identifying the difference between gut microbiota of the healthy people and CRC patients is fundamental to understand these microbes' functional roles in the development of CRC. We studied the microbial community structure of a CRC metagenomic dataset of 156 patients and healthy controls, and analyzed the diversity, differentially abundant bacteria, and co-occurrence networks. We applied a modified zero-inflated lognormal (ZIL) model for estimating the relative abundance. We found that the abundance of genera: *Anaerostipes, Bilophila, Catenibacterium, Coprococcus, Desulfovibrio, Flavonifractor, Porphyromonas, Pseudoflavonifractor*, and *Weissella* was significantly different between the healthy and CRC groups. We also found that bacteria such as *Streptococcus, Parvimonas, Collinsella, and Citrobacter* were uniquely co-occurring within the CRC patients. In addition, we found that the microbial diversity of healthy controls is significantly higher than that of the CRC patients, which indicated a significant negative correlation between gut microbiota diversity and the stage of CRC. Collectively, our results strengthened the view that individual microbes as well as the overall structure of gut microbiota were co-evolving with CRC.

## Introduction

A large number of microbes colonize the human body. They form a complex microbial community, or microbiota (Tringe et al., 2005; Zhao et al., 2013; Liao et al., 2015). Among them, the gut microbiota is the most diverse, with more than 1,000 species (Kostic et al., 2012; Li et al., 2012; Ahn et al., 2013). Those microbes are involved in maintaining intestinal homeostasis, through physiological processes such as metabolism, immune responses, and inflammation, all of which are essential for human health. Previous studies revealed a deliciated and dynamic balance between the microbial community and the host, which is likely the result of long term co-evolution. However, studies also observed that pathogenic changes in the structure, composition, and function of gut microbiota can lead to various diseases, often by causing the production of abnormal metabolites (Chen et al., 2016a; Huang et al., 2017a,b). Those diseases and conditions include irritable bowel syndrome (Kipanyula et al., 2013), Crohn's disease (Sommer and Bäckhed, 2013), and colorectal cancer (CRC) (Zackular et al., 2014; Rea et al., 2018).

The mechanisms by which gut microbes influence the CRC tumorigenesis (Iacob et al., 2017) were actively under study. For examples, researchers have recently learned that the gut microbiota plays a regulatory role in the tumor microenvironment and thus in tissue carcinogenesis (Sohn et al., 2015; Nagy-Szakal et al., 2017; Morgillo et al., 2018). Guo et al. also found that the microbiota structure and microbial metabolites can affect the body's susceptibility to CRC by directly inducing pathological conditions, such as adenoma (Guo et al., 2015). However, to further understand such interactions, it is essential to characterize and compare the gut microbiota structure of healthy controls and cancer patients. And based on that, specific microbiota patterns or strain types need to be identified to provide new targets and strategies for cancer prevention and treatment (Hu et al., 2017, 2018; Zhao et al., 2018a,b,c). Therefore, in this paper, we aim to determine the microbes that are associated with CRC using a large-scale metagenomic data set.

While the metagenomics research has provided enormous scientific data for investigating the role of the gut microbiota in the context of cancer development and progression (Zhang et al., 2014), appropriate bioinformatics and statistical analyses are also required to accurately identifying the differential microbes. Several algorithms using either parametric or non-parametric tests have been proposed to determine such species. For examples, Abusleme et al. (2013) combined the Kruskal-Wallis test with the Wilcoxon rank-sum test to analyze periodontitis data and used linear discriminant analysis to identify the species with significant differences between periodontitis patients and healthy controls. Nagy-Szakal et al. used the non-parametric Mann-Whitney *U* test with Benjamini-Hochberg correction to show that the microbial composition in the intestines of patients with chronic fatigue syndrome differed significantly from that of healthy individuals (Nagy-Szakal et al., 2017). And Peng et al. conducted beta regression on the abundance of microbes to obtain regression coefficients (Peng et al., 2016).

One particular difficulty associated with the statistical testing of differential abundance is the under-sampling or dropout (Hughes et al., 2001) of less abundant microbes caused by an insufficient sequencing depth. This fact creates many zeros in the abundance values and leads to inaccurate differential analysis when only conventional normalization was applied. This issue might be mitigated with the Zero-inflated Negative Binomial modeling (ZINB) (Ridout et al., 1998). The method is now widely adopted. For examples, Paulson et al. analyzed the differential abundance in sparse high-throughput large-scale microbial marker gene survey data by using a zero-inflated Gaussian distribution mixture model with cumulative-sum scaling normalization (Paulson et al., 2013). Zhang et al. (2016) identified differentially abundant taxa between two or more populations by using a ZINB regression method and estimated the model parameters by Expectation Maximization algorithm. Chen et al. proposed a zero-inflated Beta regression model which included two parts: a logistic regression component and a Beta regression component, for testing the association between microbial abundance and clinical covariates for longitudinal microbiome data (Chen and Li, 2016). Chen Jun et al. in 2017, proposed a robust and powerful framework of differential analysis of microbiome data based on a zero-inflated negative binomial (ZINB) regression model (Chen et al., 2017). They also proposed an omnibus test of all the parameters. Omnibus test was compared with previous methods [edgeR (Robinson et al., 2010), RAIDA (Sohn et al., 2015), DESeq2 (Love et al., 2014), and metagenomeSeq (Paulson et al., 2013)] by using simulated data. RAIDA had slightly worse FDR control at a high nominal level than omnibus test, but better FDR control than other methods. The performance of RAIDA was close to that of the omnibus test, and were higher than one of other methods. RAIDA is more effective at controlling FPR than other method including the omnibus test.

In this study, we identified the differentially abundant gut microbes between CRC and healthy samples using the *Ratio Approach for Identifying Differential Abundance* (RAIDA) algorithm (Sohn et al., 2015). The algorithm fitted the distribution of observed data with a modified zero-inflated lognormal (ZIL) model and estimated the statistical significance of abundance difference by the *T*-test. Furthermore, we used the GRAMMy algorithm (Xia et al., 2011) to estimate and analyze the relative abundance of gut microbes and diversity of the microbial communities. Finally, we constructed and analyzed a microbial association network based on all healthy, small adenoma, large adenoma, and CRC samples.

## Materials and Methods

### Two Metagenomics Datasets

Our first gut metagenomics dataset was downloaded from the European Nucleotide Archive (ENA) database (accession number ERP005534) (Table 1). The dataset (Zeller et al., 2014) consists of 156 samples from France (61 healthy, 27 small adenoma, 15 large adenoma, and 53 CRC samples). Samples with an adenoma diameter smaller than 10 mm were classified as small adenoma while those with larger than 10 mm ones were classified as large adenoma.

**Table 1:** Number of experimental samples.

**Total number of samples**	**Healthy control**	**Adenoma**		**Colorectal cancer**			
		**Small (<1 cm)**	**Large (>1 cm)**	**Early stage**		**Late stage**	
				**I**	**II**	**III**	**IV**
156	61	27	15	15	7	10	21

Our second gut metagenomics dataset was also downloaded from the ENA database (accession number ERP008729) (Zeller et al., 2014). The dataset included 156 samples from Austria, including 63 healthy samples, 47 adenoma patient samples, and 46 CRC patient samples.

### A Modified ZIL Model

We estimated the relative abundance of gut microbes using the GRAMMy algorithm. We then identified differentially abundant microbes by the RAIDA algorithm which uses a modified ZIL model to account for ratios with zeros. Metagenomic data are typically sparse because of undersampling of the microbial community or insufficient sequencing depth. The resulting abundance table is over-presented with zeros assumed that most of those zeros is a result of insufficient sequencing depth, i.e., the under-sampling of the microbial community. Based on the assumption that most microbes are not differentially abundant, the RAIDA algorithm was systematically demonstrated to consistently identify differentially abundant microbes. We adapted the RAIDA model for our statistical analysis as follows.

Let γ_ij_ denote the observed count for microbes *i* and sample *j*, and let *r*_*ij*_ denote the ratio of γ_ij_ to γ_*kj*_, where *k* represents the microbe (or a set of microbes) used as a divisor and γ_*kj*_ > 0 for all *j*. Here, *i* = 1, 2, …, *n* and *j* = 1, 2, …, *m*. The abundance ratio computed this way is denoted as $R_{ij}^{\varepsilon }$ such that:

(1) $$R_{ij}^{\varepsilon }\sim \left\{ \begin{array}{l} Unif\left(0,\varepsilon \right)\;with\;probability\;p_{i}\\ LN\left(\mu_{i},\sigma_{i}^{2}\right)\;with\;probability\text{ 1}-p_{i} \end{array}\right.$$

In this study, we used ε = min(*r*_*ij*_|*r*_*ij*_ > 0) for all *i* and *j*. The parameters θ_*i*_ = (α_*i*_, μ_*i*_, σ_*i*_) were estimated by the following expectation-maximization (EM) algorithm. Given that a ratio R follows a lognormal distribution, thus:

(2) $$\begin{array}{l} LN\left(r|\mu ,\sigma^{2}\right)=\frac{1}{\sigma \sqrt{2\pi r}}\exp\left[-\frac{{\left(\log r-\mu \right)}^{2}}{2\sigma^{2}}\right], \end{array}$$

in which, by definition, *Y* = log*R* is normally distributed with mean μ and variance σ^2^. Let $y_{ij}=\log r_{ij}^{\varepsilon }$, *z*_*ij*_ is an unobservable latent variable that accounts for the probability of zero coming from the false state. Thus, the maximum-likelihood estimate of θ_*i*_ for the modified ZIL model, i.e., Equation (1), can be obtained by solving

(3) $$\begin{array}{l} \begin{array}{c} \ell \left(\theta_{i}|y_{ij},z_{ij}\right)=\overset{m}{\underset{j=1}{\sum }}z_{ij}\log\left[\eta_{i}+\left(1-p_{i}\right)\phi \left(y_{ij};\mu_{i},\sigma_{i}^{2}\right)\right]\\ \;+\overset{m}{\underset{j=1}{\sum }}\left(1-z_{ij}\right)\log\left(1-p_{i}\right)\\ \;+\overset{m}{\underset{j=1}{\sum }}\left(1-z_{ij}\right)\log\phi \left(y_{ij};\mu_{i},\sigma_{i}^{2}\right), \end{array} \end{array}$$

where ϕ is the probability density function of a normal distribution.

### Diversity Analysis

To analyze microbial diversity, alpha diversity was used to measure the differences in gut microbial structure in the following three stages: healthy, adenoma (small and large combined), and cancer. We used the Shannon diversity index to measure the alpha diversity of the gut community. The Shannon index is defined as

(4) $$\begin{array}{l} H=-\overset{N}{\underset{j=1}{\sum }}a_{j}\ln\;a_{j}, \end{array}$$

where *H* represents the Shannon Index, *N* indicates the total number of microbial species detected, and *a*_*j*_ indicates the relative abundance of the *j* th microorganism.

## Results and Discussion

### Alpha Diversity of Gut Microbiota Predicts Colorectal Cancer Status

**Figure 1:**
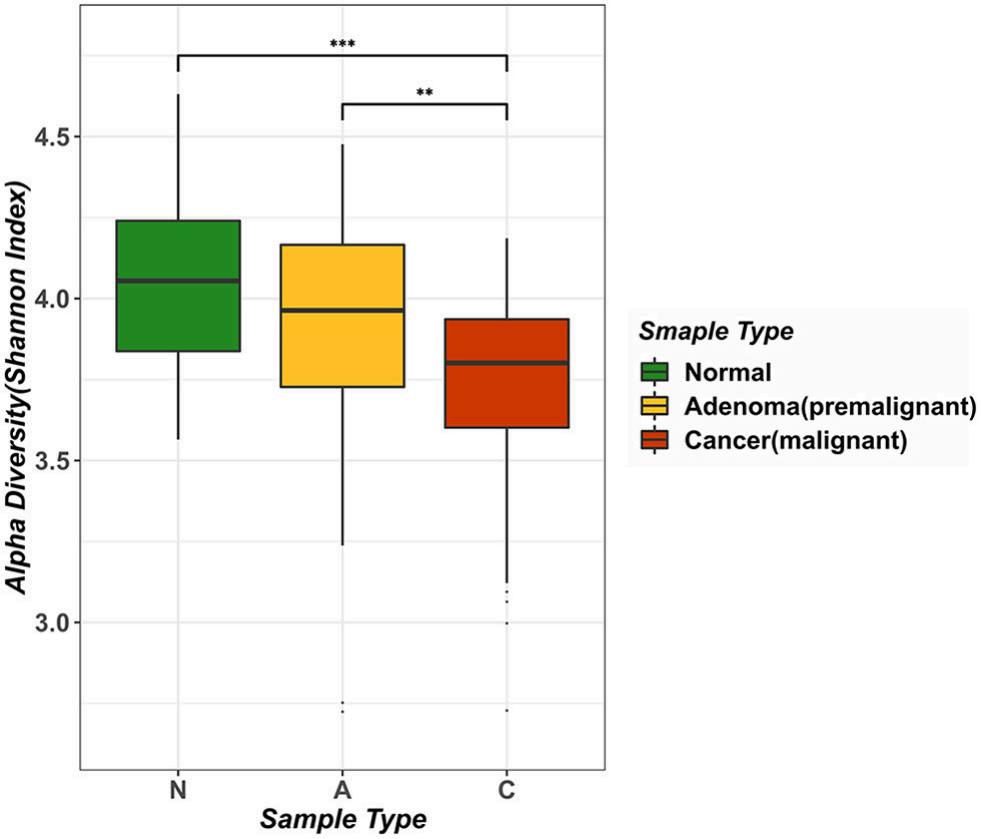
Analysis of intestinal microbial diversity in different environments. The three colors in the figure indicate the microbial diversity in different states: green represents the healthy samples, yellow represents adenoma (precancerous lesion) growth in the intestine, and red represents a sample of colorectal cancer patients. The average value of Alpha diversity of healthy samples was 4.0456, whereas the counterpart in the adenoma sample was 3.8957, and that in the cancer sample was 3.7161.

We computed the alpha diversity of gut microbes of the healthy samples, adenoma samples and CRC samples using the Shannon index and compared them with the rank-sum Dunn test (Figure 1). We found that the alpha diversity was significantly lower in the CRC samples as compared to the healthy samples (two tailed, Dunn test, *P* < 0.0001) and adenoma samples (two tailed, Dunn test, *P* = 0.0021). However, the alpha diversity of the healthy and adenoma samples was not significantly different (two tailed, Dunn test, *P* = 0.0571). To study the relationship between the probability of cancer occurrence and the alpha diversity, we performed logit regression to associate CRC status with the Shannon index. The regression results showed that the Shannon index is a significant predictor of CRC status (univariate logistic model, *P* < 0.05). The fitted logistic regression model was as follows:

(5) $$\begin{array}{l} P=\frac{\exp\left(-4.563d+17.546\right)}{1+\exp\left(-4.563d+17.546\right)}, \end{array}$$

i.e., logit(*P*) = −4.563*d* + 17.546, where *P* is the probability of being CRC, and *d* is the Shannon diversity index. We provided the plot of the relationship of probability of cancer occurrence and Shannon index of adenoma patients as show in Figure S1. Our result suggested that the diversity of the microbial species in the human intestines decreases as colorectal malignancies grow, which was supported by literature (Ahn et al., 2013).

### Nine Genera Were Differentially Abundant in the Colorectal Cancer Gut Environment

**Figure 2:**
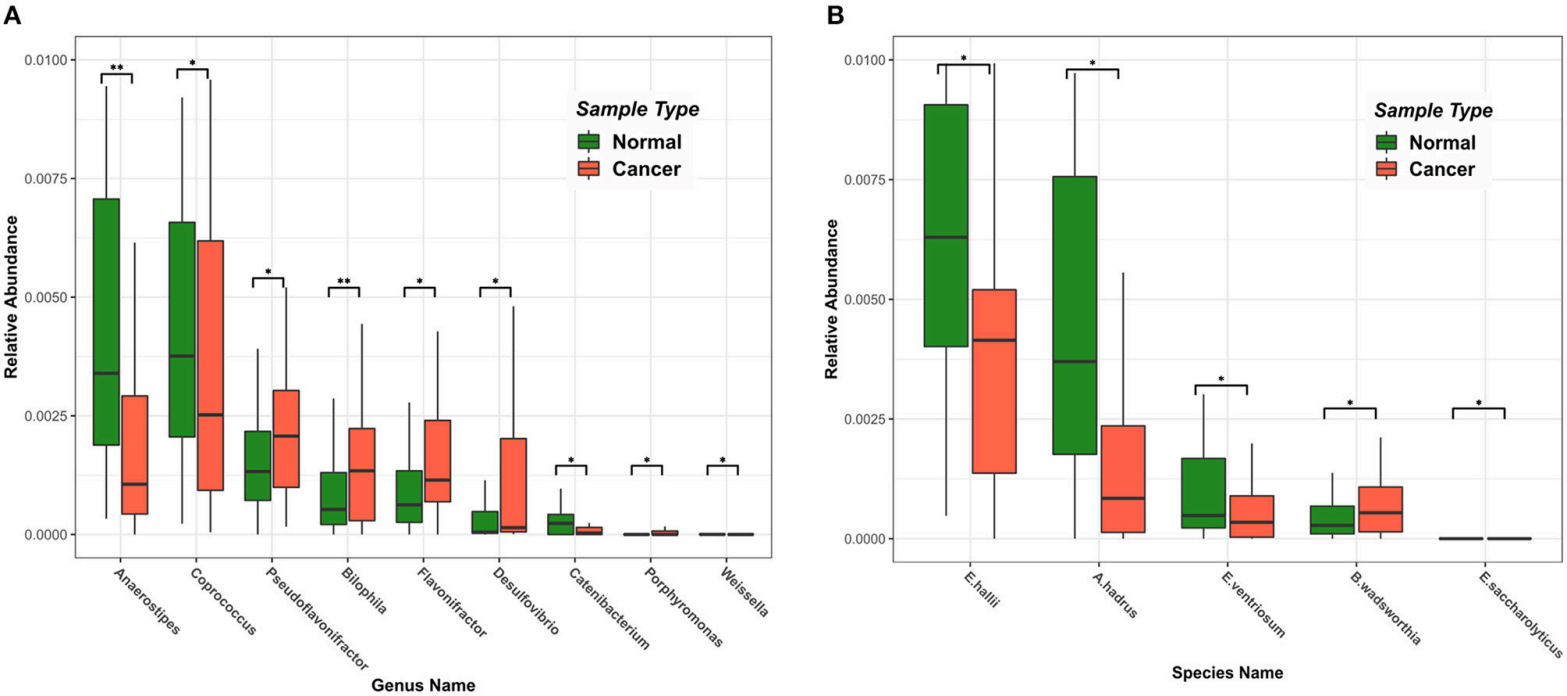
Differences in intestinal microbiome at the genus and species level among samples of different states. Green represents a healthy sample, and red represents a colorectal cancer sample. **(A)** The top nine microbial genera with significant differences in abundance. **(B)** The top five microbial strains with significant differences in abundance.

Using the RAIDA algorithm, we identified nine microbial genera that were significantly different in abundance between the CRC and the controls, which included *Anaerostipes, Coprococcus, Pseudoflavonifractor, Bilophila, Flavonifractor, Desulfovibrio, Catenibacterium, Porphyromonas*, and *Weissella* (Figure 2A). We first observed that the abundance of *Coprococcus* was higher in the healthy samples as compared to the CRC patients. As a validation, Shen et al. showed that colorectal adenomas had lower relative abundance of *Bacteroides* spp. and *Coprococcus* spp. than controls (Shen et al., 2010). The metabolic activity of butyrate-producing bacteria is the major source of butyrate in human body. *Coprococcus* is among the essential butyrate-producing genera in human body, which promote colonic health by mediating anti-inflammatory and antitumor effects, as well as providing energy for colonocytes (Singh et al., 2014).

Also notable in our result were the genera *Fusobacterium (Fusobacteriaceae)* and *Porphyromonas (Porphyromonadaceae)*, which were shown highly enriched in the CRC patients. So was the species *Bibliophile wadsworthia*. Those sulfidogenic bacteria, including *Desulfovibrio, Fusobacterium*, and *Bilophila wadsworthia*, likely participate in the development of CRC by producing hydrogen sulfide (Ridlon et al., 2016; Dahmus et al., 2018). *Bilophila wadsworthia* was additionally reported to cause systemic inflammatory response in a preclinical mice study (Zhou et al., 2017).

Interestingly, we also observed that the abundance of *Eubacterium hallii, Anaerostipes hadrus*, and *Eubacterium ventriosum* (Figure 2B) were significantly higher in the healthy samples than in the CRC samples. *E. hallii* and *A. hadrus* can utilize the glucose and fermentation intermediates acetate and lactate to form butyrate and hydrogen, which were considered important microbes in maintaining intestinal metabolic balance (Christina et al., 2016).

We also found that *Flavonifractor* was higher in the healthy samples than that in the CRC samples, which was in agreement with Anand et al. (2016). We also observed that *Anaerostipes* had a significantly lower abundance in the CRC samples, which agreed with previous studies (Peters et al., 2016; Mori et al., 2018). We found that no *Catenibacterium* and *Gardnerella* (*Bifidobacteriaceae*) were present in CRC patient samples, which was supported by Chen et al. (2012).

We tested if the nine differentially abundant genera are viable biomarkers to distinguish healthy individuals from CRC patients. We trained a random forest classifier using a 5-fold cross-validation (rotative using 80% data as the training set the rest 20% as the testing set) using the first metagenomic dataset. The classifier achieved an Area Under Curve (AUC) of 0.9333.

### Microbial Co-occurrence Network Evolves With CRC Development

**Figure 3:**
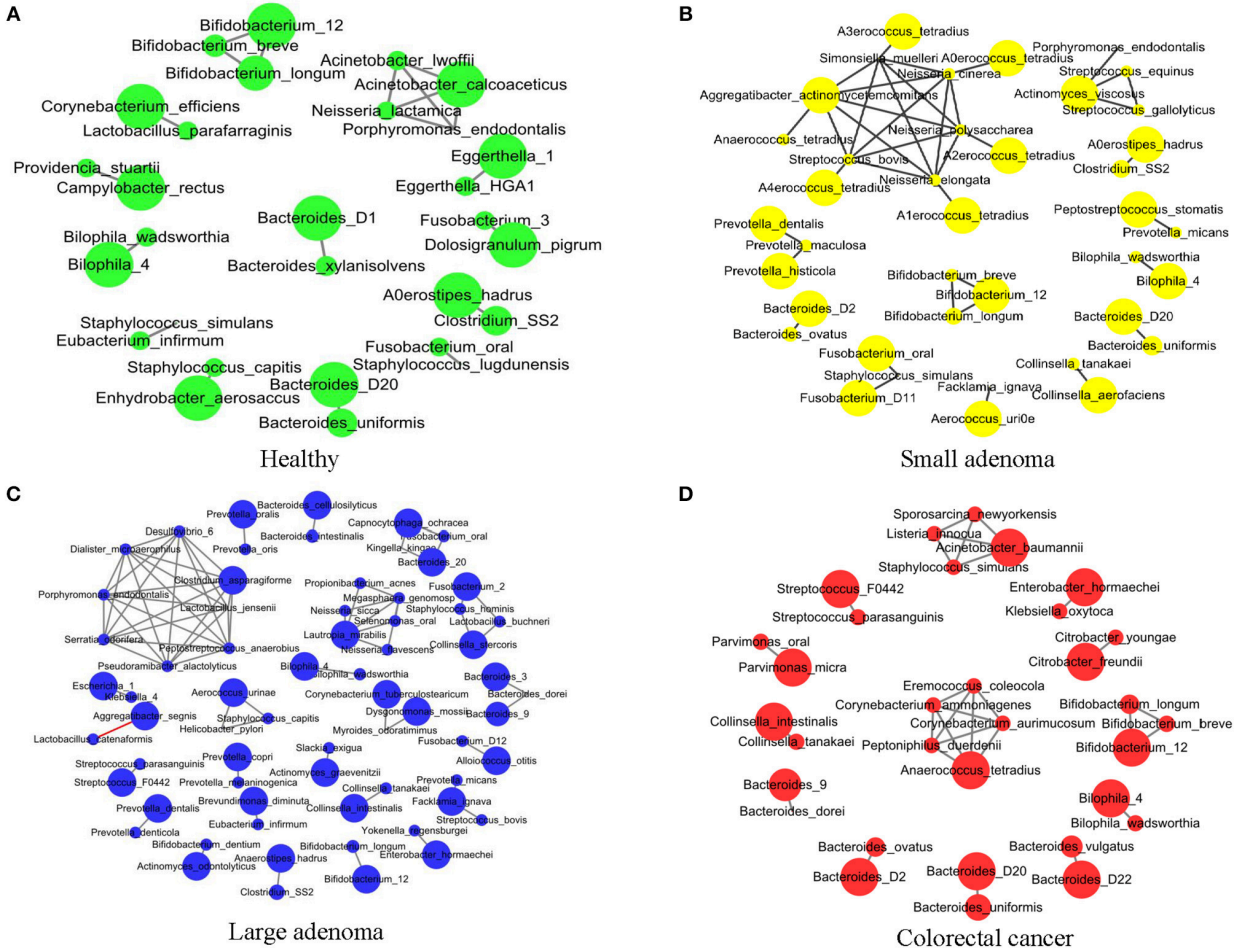
The association network of intestinal microbiome in different states. Each circle represents the average relative abundance of a microbial species in that state. The higher the average relative abundance, the larger the area of the circle. The solid gray line between the circles indicates a positive Spearman correlation between the two groups, and the solid red line indicates a negative Spearman correlation between the two groups. **(A–D)** The association network of intestinal microbiome in healthy, small adenoma, large adenoma and cancer samples.

Sophie Weiss et al. compared 8 methods of establishing association networks, they recommend filtering out extremely rare OTUs prior to network construction (Weiss et al., 2016). According to Figure 7 in this paper, SparCC should be used when the inverse simpson n_eff_ of microbes < 13, SparCC maintain high precision compared with predictions on abundance tables with low n_eff_. But the inverse simpson n_eff_ of microbes is 27.9 (>13) in our paper, abundance of OTUs are more than 50% sparse. So we calculated the correlation between species by Pearson correlation coefficient (Pearson, 1909). We further conducted an association network analysis to identify the co-occurring intestinal microbes under different CRC states. All significant co-occurrences (PCC > 0.5) were found to be within the same genera, such as *Bifidobacterium, Bacteroides*, and *Bilophila* (Figure 3). Furthermore, both *Bifidobacterium* and *Bacteroides* were previously identified by us to have significant differences in abundance between healthy controls and CRC patients (Figure 3A). It is thus reasonable to assess that these bacteria were pathogenic as a group because the change of abundance in one them can result in changes of abundance in the entire clique. Our observation supported the theory that CRC ensues an interrupted balance between these bacteria (Brennan and Garrett, 2016; Yazici et al., 2017).

Co-occurrence was also found among species of the genus *Prevotella* in the healthy, small adenoma, and large adenoma environments (Figures 3A–C), however, such co-occurrence was missing in the CRC environment (Figure 3D). Conversely, several species of the genera *Streptococcus, Parvimonas, Collinsella*, and *Citrobacter* were only co-occurring in the cancer environment. Overall, we observed fewer microbial co-occurrences the healthy environment. While, in the adenoma environments, we found an increase of co-occurring pathogenic microbes. The number of co-occurring microbes was then reduced in the CRC environment. The total number of co-occurrence is relatively close between the healthy and the CRC environment, however, the microbes involved were distinct. The number of total co-occurrence might have peaked at the adenoma environments because of the co-existence of competing homeostatic and pathogenic microbial interactions in the intermediacy stage.

## Conclusions

We analyzed the alpha diversity of the gut microbial community of 156 healthy, adenoma and CRC samples. We found the alpha diversity was significantly higher in healthy samples as compared to the CRC samples. We applied a modified ZIL model and identified nine significantly different genera between the healthy and CRC groups, i.e., *Anaerostipes, Bilophila, Catenibacterium, Coprococcus, Desulfovibrio, Flavonifractor, Porphyromonas, Pseudoflavonifractor*, and *Weissella*. We used these nine genera as input features for a random forest classifier and successfully predicted the CRC status with a high AUC score of 0.9333. Our results suggested that the community member and the overall structure of the gut microbiota are potential effective biomarkers of CRC stages. This avenue is being actively pursued by us and other computational researchers (Chen and Yan, 2013; Chen et al., 2016b,c, 2018a,b,c; Chen and Huang, 2017), who may bring in novel strategies for preventing and curing CRC in the near future.

## Author Contributions

DA and YG conducted the analysis, summarized the result and drafted the manuscript. HP, XL, and GL assisted in the data analysis and contributed to the manuscript. DA and LX conceived the study. LX supervised the manuscript writing. All authors have read and approved the final manuscript.

### Conflict of Interest Statement

The authors declare that the research was conducted in the absence of any commercial or financial relationships that could be construed as a potential conflict of interest.

## Supplementary Material

Figure S1 Logit regression prediction results of the Shannon diversity index. The blue circle in the figure represents a large adenoma sample, and the red triangle represents a small adenoma sample.
