# Supplementary figures and images for: Identifying Gut Microbiota Associated With Colorectal Cancer Using a Zero-Inflated Lognormal Model
Source: Front Microbiol. 2019 Apr 24;10:826. doi: 10.3389/fmicb.2019.00826 (PMC6491826; doi:10.3389/fmicb.2019.00826)

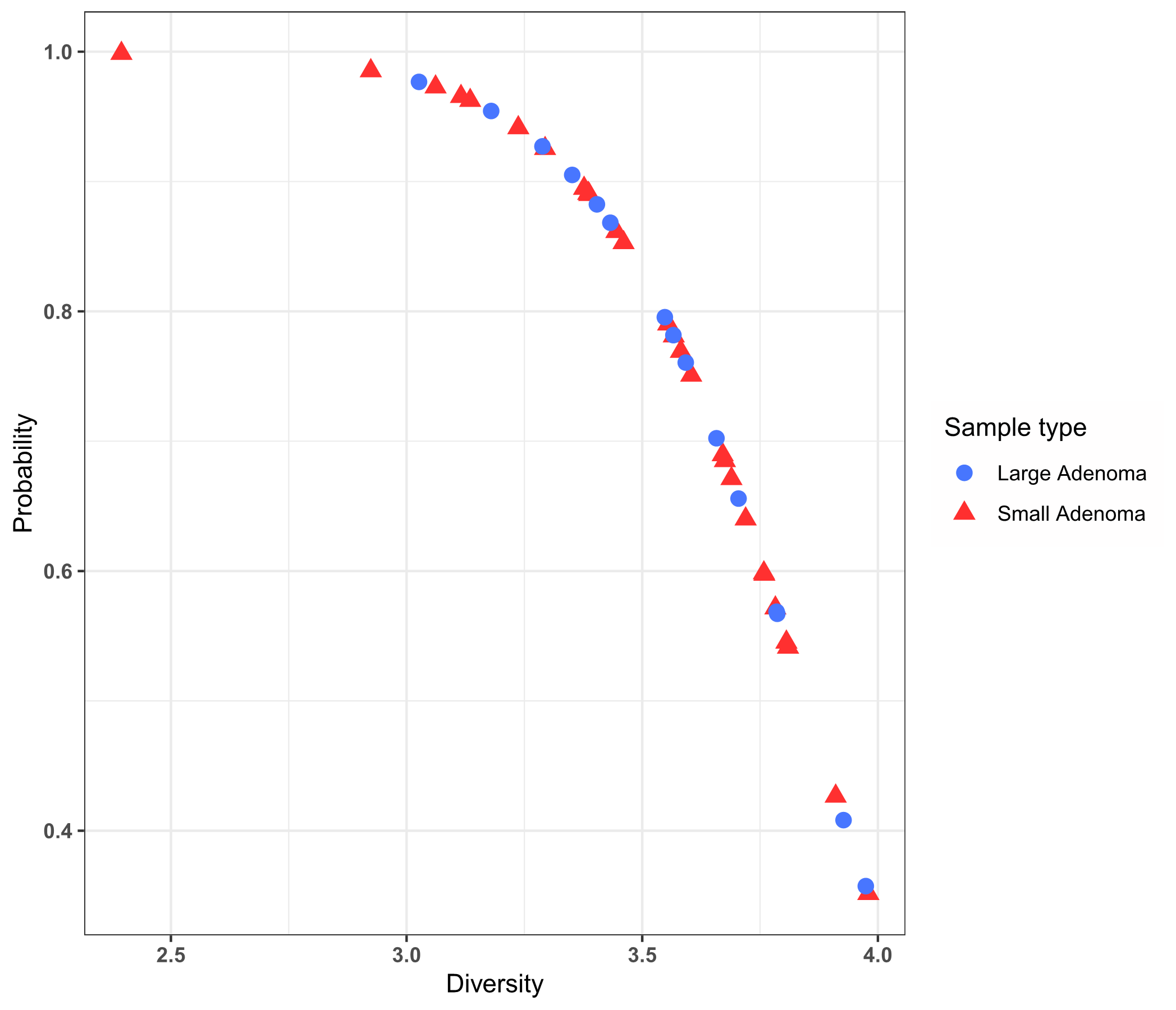

Supplement: Figure S1 — Logit regression prediction results of the Shannon diversity index. The blue circle in the figure represents a large adenoma sample, and the red triangle represents a small adenoma sample. [file Image_1.TIF]
